# Characterization of the Immune-Modulating Properties of Different β-Glucans on Myeloid Dendritic Cells

**DOI:** 10.3390/ijms25189914

**Published:** 2024-09-13

**Authors:** Hannah Rainer, Alexandra Goretzki, Yen-Ju Lin, Hannah Ruth Schiller, Maren Krause, Sascha Döring, Daniel Strecker, Ann-Christine Junker, Sonja Wolfheimer, Masako Toda, Stephan Scheurer, Stefan Schülke

**Affiliations:** 1 Section Molecular Allergology, Paul-Ehrlich-Institut, 63225 Langen, Germany; 2 Section Research Allergology, Division of Allergology, Paul-Ehrlich-Institut, 63225 Langen, Germany; 3 Laboratory of Food and Biomolecular Science, Graduate School of Agricultural Science, Tohoku University, Sendai 980-8576, Japan

**Keywords:** β-glucan, adjuvants, immune metabolism, AIT

## Abstract

In allergen-specific immunotherapy, adjuvants are explored for modulating allergen-specific Th2 immune responses to re-establish clinical tolerance. One promising class of adjuvants are β-glucans, which are naturally derived sugar structures and components of dietary fibers that activate C-type lectin (CLR)-, “Toll”-like receptors (TLRs), and complement receptors (CRs). We characterized the immune-modulating properties of six commercially available β-glucans, using immunological (receptor activation, cytokine secretion, and T cell modulating potential) as well as metabolic parameters (metabolic state) in mouse bone marrow-derived myeloid dendritic cells (mDCs). All tested β-glucans activated the CLR Dectin-1a, whereas TLR2 was predominantly activated by Zymosan. Further, the tested β-glucans differentially induced mDC-derived cytokine secretion and activation of mDC metabolism. Subsequent analyses focusing on Zymosan, Zymosan depleted, β-1,3 glucan, and β-1,3 1,6 glucan revealed robust mDC activation with the upregulation of the cluster of differentiation 40 (CD40), CD80, CD86, and MHCII to different extents. β-glucan-induced cytokine secretion was shown to be, in part, dependent on the activation of the intracellular Dectin-1 adapter molecule Syk. In co-cultures of mDCs with Th2-biased CD4^+^ T cells isolated from birch allergen Bet v 1 plus aluminum hydroxide (Alum)-sensitized mice, these four β-glucans suppressed allergen-induced IL-5 secretion, while only Zymosan and β-1,3 glucan significantly suppressed allergen-induced interferon gamma (IFNγ) secretion, suggesting the tested β-glucans to have distinct effects on mDC T cell priming capacity. Our experiments indicate that β-glucans have distinct immune-modulating properties, making them interesting adjuvants for future allergy treatment.

## 1. Introduction

β-glucans are biologically active polysaccharides consisting of glucose monomers (linked in β-conformation) with varying degrees of branching [1]. β-glucans can be derived from different organisms such as yeast, fungi, bacteria, grain, or seaweed [2]. Their structural characteristics, including linkage type, degree of branching, and conformation of glucan chains, depend on their origin, and it is suggested that they determine their physiochemical, biological, and immunological properties [2,3,4]. β-glucans possess immune-modulatory properties, including the potential to induce trained immune responses [5,6] and anti-microbial activity, to promote phagocytosis in macrophages and neutrophils [7] and to trigger the secretion of the pro-inflammatory cytokines IL-1β, IL-6, and TNFα [8,9]. β-glucans mediate these effects by activating different pattern recognition receptors (PRRs), including the C-type lectin receptor (CLR) Dectin-1 [6,10], “Toll”-like receptor 2 (TLR2) [11], and the complement receptor 3 [12]. Accordingly, β-glucans can trigger different intracellular signaling pathways. Therefore, they may be utilized as novel adjuvants for the prevention and treatment of various diseases [1,13,14].

In this context, a better understanding of the mechanisms underlying the regulation of immune cell effector functions by allergen therapeutics, vaccines, and adjuvants, which induce trained immune responses (such as β-glucans), would be highly beneficial in the development of novel therapeutics and vaccines, combining trained immune responses with the induction of classical antigen-specific adaptive immune responses [15].

In this study, we analyzed the immunological properties of six different commercially available β-glucan preparations of varying composition: Zymosan and Zymosan depleted (Zymosan depl.), Curdlan, Laminarin, as well as two previously investigated β-glucans [16]—β-1,3 glucan and β-1,3 1,6 glucan—which were both derived from *Saccharomyces cerevisiae*. Zymosan is a *Saccharomyces cerevisiae*-derived β-1,3-linked glucan, which was described to activate Dectin-1 as well as TLR2, whereas, for its variant, Zymosan depl., hot alkali treatment abrogates TLR2-activation, resulting in activation of only Dectin-1. Curdlan is a mainly β-1,3-linked glucan, which also contains some intra- or interchain β-1,3 1,6 linkages isolated from the bacterium *Alcaligenes faecalis* that activates Dectin-1 [17,18,19]. Laminarin derived from the brown seaweed *Laminaria digitata* is a linear β-1,3 glucan with β-1,6 branches, which was described to either act as an agonist or antagonist to Dectin-1 [20,21,22,23]. Finally, *Saccharomyces cerevisiae*-derived β-1,3 glucan and β-1,3 1,6 glucan preparations, which were previously investigated for their capacity to prevent allergic reactions [16], were also included.

Our analyses comprised receptor activation studies and a direct comparison of the six β-glucans regarding their immune-modulating properties on C57BL/6 mouse myeloid dendritic cells (mDCs). Furthermore, we studied the effect of Zymosan-, Zymosan depl.-, β-1,3 glucan-, or β-1,3 1,6 glucan-stimulated mDCs on their T cell priming capacity in vitro, using BALB/c mDC:T cell co-cultures.

## 2. Results

### 2.1. β-Glucans Differentially Activate mDCs In Vitro

According to the solubility of the tested β-glucans, their concentration was adjusted to 10 mg/mL in either phosphate-buffered saline (PBS, Repository Figure S1A) or PBS 0.1 M NaOH. β-glucan preparations were inspected visually for solubility (Repository Figure S1A). The addition of 0.1 M NaOH slightly impaired β-glucan solubility. In addition, to exclude the cytotoxic effects of NaOH in the used cell culture system, all mDC stimulations were performed with β-glucans solved in PBS.

β-glucans were checked for the presence of proteins by SDS-PAGE (Repository Figure S1B). Here, Zymosan, and to a lesser extend Zymosan depl., contained detectable proteins (sizes of approximately 10 kDa, 110 kDa, and proteins that were not able to enter the stacking gel, Repository Figure S1B).

We furthermore determined the endotoxin levels of the tested β-glucans via the PTS LAL-test (Repository Figure S1C). Endotoxin levels were lowest for β-1,3 glucan (2.28 pg/µg β-glucan), Laminarin (8.6 pg/µg β-glucan), and Zymosan depl. (7.98 pg/µg β-glucan); medium levels were detected for Zymosan (50.2 pg/µg β-glucan), and rather high levels for β-1,3 1,6 glucan (347 pg/µg β-glucan) and Curdlan (15,400 pg/µg β-glucan, Repository Figure S1C). Furthermore, Zymosan (142.3 µg/mL), Zymosan depl. (95.8 µg/mL), Laminarin (150.8 µg/mL), and β-1,3 1,6 glucan (38.9 µg/mL) contained detectable levels of glucose (Repository Figure S1D), while BCA and Bradford assay detected protein levels between 24.7 µg/mL (Curdlan) and 463.7 µg/mL (Zymosan, Repository Figure S1E).

**Figure 1:**
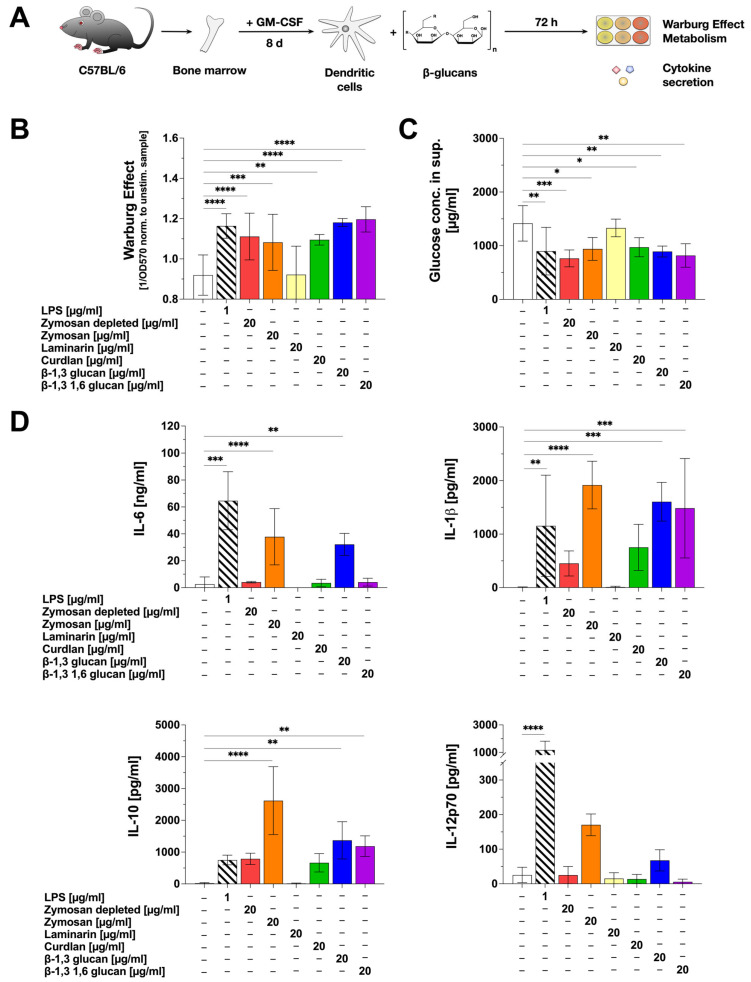
β-glucans differ in their activation of mDC metabolism and the secretion of pro- and anti-inflammatory cytokines. Bone marrow of C57BL/6 mice was isolated, differentiated into mDCs for 8 days, and subsequently stimulated with either 20 µg/mL of the indicated β-glucans or 1 µg/mL of LPS as a positive control (**A**). The Warburg Effect was measured at OD_570nm_, and the inverted values were normalized to the unstimulated controls (**B**). The glucose concentration in the cell culture supernatant was determined using the Glucose (GO) assay kit and measuring the absorption at OD_540nm_ (**C**). The secretion of the cytokines IL-6, IL-1β, IL-10, and IL-12p70 was measured via sandwich ELISA at OD_450nm_. Data are mean results ± SD of three independent experiments (**D**). Statistical comparison was performed by 1-way ANOVA with correction for multiple comparisons according to Dunnett and indicated as follows: no indication = not significant *p*-value > 0.05, * *p*-value < 0.05, ** *p*-value < 0.01, *** *p*-value < 0.001, or **** *p*-value < 0.0001.

Next, we investigated the potential of the tested β-glucans to activate mDC metabolism and cytokine secretion using C57BL/6 bone-marrow-derived mDCs (Figure 1A). In this experimental setup, except for Laminarin, all tested β-glucans induced a significant Warburg Effect in comparison to the unstimulated control (Figure 1B). The Warburg Effect results from a predominant production of lactate from glucose via glycolysis. Accordingly, glucose concentrations were decreased by approx. 33% upon stimulation with the β-glucans that induced a Warburg Effect (all tested β-glucans except Laminarin) (Figure 1C).

Moreover, the β-glucans differentially induced the secretion of both pro- and anti-inflammatory cytokines (Figure 1D). Pro-inflammatory IL-6 secretion was induced by Zymosan and β-1,3 glucan, whereas only low IL-6 levels, comparable to the unstimulated control, were detected upon stimulation with Zymosan depl., Laminarin, Curdlan, and β-1,3 1,6 glucan (Figure 1D). Secretion of the pro-inflammatory cytokine IL-1β was significantly induced by Zymosan, β-1,3 glucan, and β-1,3 1,6 glucan. Zymosan depl. and Curdlan also increased IL-1β levels, which were, however, not significantly different from the unstimulated control (Figure 1D). Significant levels of the anti-inflammatory cytokine IL-10 were detected after stimulation of mDC cultures with Zymosan, β-1,3 glucan, and β-1,3 1,6 glucan, but not with Zymosan depl., Laminarin, or Curdlan (Figure 1D). Here, stimulation with 20 µg/mL Zymosan resulted in high levels of IL-10 secretion (1.9 ng/mL), being about three times higher than for the positive control LPS (stimulation conc. 1 µg/mL: 712 pg/mL) (Figure 1D). A secretion of the pro-inflammatory cytokine IL-12p70 was only induced by Zymosan and β-1,3 glucan (Figure 1D).

In summary, stimulation of mDCs with Zymosan led to the highest secretion levels of all tested cytokines. Β-1,3 glucan induced the secretion of IL-6, IL-1β, and IL-10, whereas β-1,3 1,6 glucan only resulted in the secretion of IL-1β and IL-10. In contrast, Laminarin and Curdlan did not induce a statistically significant secretion for any of the tested cytokines.

### 2.2. Stimulation of mDCs with β-Glucans Results in Increased Metabolic Activity

**Figure 2:**
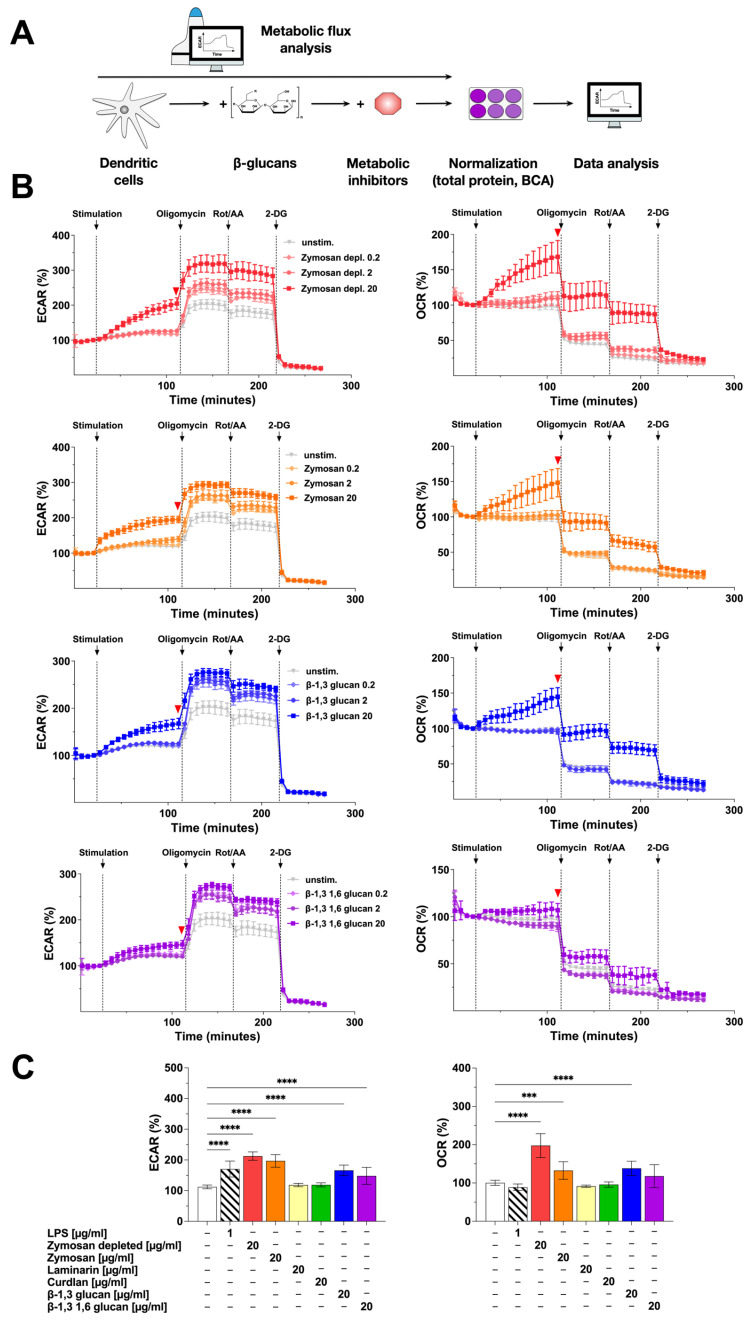
Stimulation of mDCs with β-glucans results in increased metabolic activity. Bone marrow of C57BL/6 mice was isolated, differentiated into mDCs for 8 days, and subsequently analyzed in extracellular flux assays using Agilent Seahorse technology (**A**). mDCs were seeded overnight into Seahorse XF96 cell culture microplates, stimulated with increasing doses of the indicated β-glucan for 14 cycles (84 min), and analyzed for ECAR and OCR. Afterward, ATP synthase, ETC, and glycolysis were inhibited by sequentially injecting oligomycin, Rotenone/antimycin A (Rot/AA), and 2-deoxyglucose (2-DG), respectively, for 8 cycles (48 min) each. Data are representative of three independent experiments (**B**). The red arrow indicates the measurement cycle used for statistical analysis. Data are mean results ± SD of three independent experiments (**C**). Statistical comparison was performed by 1-way ANOVA with correction for multiple comparisons according to Dunnett and indicated as follows: no indication = not significant *p*-value > 0.05, *** *p*-value < 0.001, or **** *p*-value < 0.0001. Abbreviations: ECAR: extracellular acidification rate, OCR: oxygen consumption rate, Rot/AA: rotenone/antimycin A, 2-DG: 2-deoxyglucose.

In order to correlate the observed activation of Warburg metabolism and cytokine secretion by the β-glucan-stimulated mDCs, we next investigated the metabolic phenotype of β-glucan-stimulated mDCs in more detail (Figure 2). For this, the contribution of glycolysis (measured as the extracellular acidification rate (ECAR) caused by the secretion of glycolysis-derived lactate into the cell culture medium) and mitochondrial respiration (measured as the cellular oxygen consumption rate (OCR)) to the observed mDC activation by the different β-glucans (Figure 2B,C) were investigated by extracellular flux assays using Agilent Seahorse technology (Figure 2A).

Compared to the unstimulated control, ECAR values dose-dependently increased upon stimulation of the mDCs with Zymosan depl. (increase to 213% in cycle 18 compared to unstimulated control for a stimulation concentration of 20 µg/mL), Zymosan (stimulation conc. 20 µg/mL: 197% in cycle 18), β-1,3 glucan (stimulation conc. 20 µg/mL: 166% in cycle 18), or β-1,3 1,6 glucan (stimulation conc. 20 µg/mL: 148% in cycle 18) (Figure 2B,C).

In parallel, OCR values also dose-dependently and significantly increased after stimulation with Zymosan depl. (198% in cycle 18 compared to unstimulated control for a stimulation conc. of 20 µg/mL), Zymosan (stimulation conc. 20 µg/mL: 133% in cycle 18), or β-1,3 glucan (stimulation conc. 20 µg/mL: 138% in cycle 18) (Figure 2B,C). In this experimental setting, neither stimulation with Laminarin nor Curdlan resulted in a detectable activation of mDC metabolism (Repository Figure 2C and Figure S2).

Inhibition of the ATP synthase by injection of oligomycin further increased ECAR for all tested β-glucans (Figure 2B, left) while the OCR of mDCs stimulated with 20 µg/mL of the tested β-glucans either decreased to levels observed in (Zymosan depl., Zymosan, or β-1,3 glucan) or below (β-1,3 1,6 glucan) the unstimulated control (Figure 2B, right). After additional injection of the electron transport chain (ETC) inhibitors rotenone and antimycin A (Rot/AA), the ECAR remained unchanged except for a slight drop for all concentrations of all tested β-glucans (Figure 2B, left). The OCR was further decreased by 30 to 50% compared to the last cycle before the injection of the inhibitor (Figure 2B, right). Finally, adding the glycolysis inhibitor 2-deoxyglucose (2-DG) completely suppressed ECAR as well as OCR in all tested conditions (Figure 2B).

In conclusion, the tested β-glucans differently activated mDC metabolism: Zymosan, but also Zymosan depl., and β-1,3 glucan dose-dependently increased both glycolysis and mitochondrial respiration. In contrast, the effects of β-1,3 1,6 glucan (increased glycolysis but not mitochondrial respiration), Laminarin, and Curdlan (both did not activate mDC metabolism) on mDC metabolism were less pronounced.

### 2.3. β-Glucans Differently Activate TLR2 and Dectin-1

β-glucans induce immune responses by activating different PRRs, including the C-type lectin receptor Dectin-1 [6,10] and TLR2 [11]. In order to correlate the distinctive effects of the investigated β-glucans on mDC cytokine secretion and metabolism with their capacity to signal via Dectin-1 and TLR2, the tested β-glucans were investigated for their capacity to activate both receptors in cell-based reporter assays (Repository Figures S3 and S4).

Human embryonic kidney (HEK)-293 cells stably transfected with human Dectin-1a, human Dectin-1b, or mouse TLR2 were stimulated with increasing β-glucan doses for 16 h. As a proxy for TLR2 activation, the secretion of human IL-8 was detected by ELISA. Dectin-1 activation was determined by photometrical detection of secreted embryonic alkaline phosphatase (SEAP) (Repository Figure S3A,B, dose-response curves shown as Repository Figure S4).

Except for a minimal hDectin-1b activation by Curdlan and no activation by Laminarin, dose-dependent activation of both hDectin-1a and hDectin-1b was observed for all tested β-glucans (Repository Figure S4). The strongest receptor activation resulted from stimulation with either 10 or 100 µg/mL of the respective β-glucan (Repository Figure S4). For statistical comparisons, the OD_635nm_ values were compared between the different β-glucans using stimulation concentrations of 100 µg/mL (Repository Figure S3C): to different extents, all tested β-glucans significantly activated hDectin-1a, with Zymosan resulting in slightly higher OD_635nm_ values (OD: 1.67, Repository Figure S3C) compared to Zymosan depl. (OD: 1.29), Laminarin (OD: 0.68), Curdlan (OD: 0.4), β-1,3 glucan (OD: 1.3), or β-1,3 1,6 glucan (OD: 1.5, Repository Figure S3C).

Except for Laminarin, hDectin-1b was also activated by all tested β-glucans (Repository Figure S4). Here, stimulation with 100 µg/mL of either Zymosan depl. (OD: 1.1) or β-1,3 1,6 glucan (OD: 1.05) led to a stronger receptor activation than observed for the same concentration of Zymosan (OD: 0.54), β-1,3 glucan (OD: 0.55), Laminarin (OD: 0.07), or Curdlan (OD: 0.13, Repository Figure S3B). Of note, activation levels of hDectin-1a were higher for all tested glucans than the levels observed for hDectin-1b (Repository Figure S3C).

A pronounced, dose-dependent activation of mTLR2 was induced by Zymosan, resulting in the secretion of up to 840 pg/mL IL-8 (Repository Figure S3C, dose dependency as Repository Figure S4). For Zymosan depl., β-1,3 glucan, or β-1,3 1,6 glucan, only low IL-8 levels of max. 100 pg/mL were detected (Repository Figure S3C, dose–response curves as Repository Figure S4), which were (except for Curdlan) still significantly different from levels observed upon stimulation of TLR2-negative control cells with the respective β-glucans (Repository Figure S3C).

Therefore, while all tested β-glucans activated hDectin-1 receptors, TLR2 activation was more pronounced in response to stimulation with Zymosan than for the other β-glucans.

### 2.4. Stimulation with β-Glucans Increases mDC Surface Expression of Both TLR2 and Dectin-1

**Figure 3:**
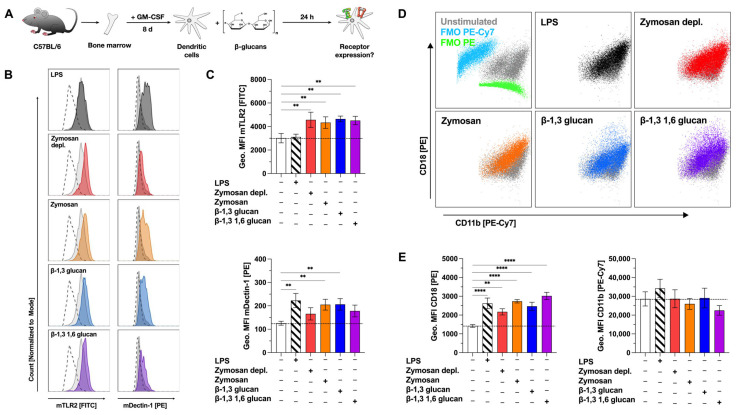
β-glucans upregulate the expression of the pattern recognition receptors TLR2 and Dectin-1 on mDCs. Bone marrow of C57BL/6 mice was isolated and differentiated for 8 days into mDCs that were subsequently stimulated with either 12 µg/mL of the indicated β-glucans or 10 µg/mL LPS as a positive control for 24 h (**A**). Cells were harvested, and surface expression of the indicated pattern recognition receptors was analyzed via flow cytometry. Stimulated samples (colored) were compared to either unstimulated controls (light grey filled) or fluorescence-minus-one (FMO)-stained cells (dashed lines). Co-expression of CD11b and CD18 forming the complement receptor 3 on LPS and β-glucan-stimulated mDCs was investigated by flow cytometry (**D**). FMOs are shown in blue for PE-Cy7 and green for PE, respectively. Data are representative results from one out of three independent experiments (**B**,**D**) or geometric mean fluorescence intensities (Geo. MFI) from three independent experiments (**C**,**E**). Dashed lines indicate the expression level of the unstimulated control. Statistical comparison was performed by 1-way ANOVA with correction for multiple comparisons according to Dunnett and indicated as follows: no indication = not significant and *p*-value > 0.05, ** *p*-value < 0.01, or **** *p*-value < 0.0001. Abbreviations: MFI: mean fluorescence intensity, FMO: fluorescence-minus-one.

After determining the capacity of the tested β-glucans to activate Dectin-1 and TLR2 (Repository Figure S3), we tested for the effect of β-glucan stimulation on Dectin-1-, TLR2-, and complement receptor 3 (CR3)-expression levels on CD11c^+^CD11b^+^B220^−^ mDCs by flow cytometry (Figure 3A). As we did not observe a pronounced cytokine secretion (Figure 1), activation of mDC metabolism (Figure 2), and receptor activation (Repository Figure S3) for either Laminarin or Curdlan, these β-glucans were excluded from further experiments.

Basal expression of both TLR2 and Dectin-1 on mDCs was investigated by comparing unstimulated samples to fluorescence-minus-one (FMO) control samples (Figure 3B, light grey filled vs. dashed lines). mDCs expressed robust levels of TLR2 and low levels of Dectin-1 compared to respective FMO controls (Figure 3B, top panels). Compared to unstimulated controls (mean fluorescence intensity (MFI): 3014), TLR2 expression was significantly increased by stimulation with Zymosan depl. (MFI: 4570), Zymosan (MFI: 4334), β-1,3 glucan (MFI: 4646), or β-1,3 1,6 glucan (MFI: 4509) (Figure 3B,C). Dectin-1 expression was significantly enhanced by stimulation with Zymosan (MFI Zymosan: 205, MFI unstimulated control: 126), or β-1,3 glucan (MFI: 207), while either Zymosan depl. (MFI: 166) or β-1,3 1,6 glucan (MFI: 178) also resulted in increased expression levels of Dectin-1, which, however, did not reach statistical significance (Figure 3C).

CR3 is a heterodimer consisting of CD11b and CD18. While the rather high expression levels of CD11b were not significantly altered by stimulation with the different β-glucans, CD18 expression levels were significantly increased by all tested stimuli compared to unstimulated cells (MFIs: unstimulated: 1422, LPS: 2623, Zymosan depl.: 2172, Zymosan: 2734, β-1,3 glucan: 2467, β-1,3 1,6 glucan: 3018, Figure 3D,E). Here, the increase in CD18 expression suggested increased expression levels of CR3 on β-glucan-stimulated mDCs.

Accordingly, the stimulation of mDCs with the tested β-glucans enhanced the mDC surface expression of TLR2, Dectin-1, and CR3.

### 2.5. β-Glucans Upregulate the Expression of Surface Activation- and Co-Stimulatory Markers on mDCs

**Figure 4:**
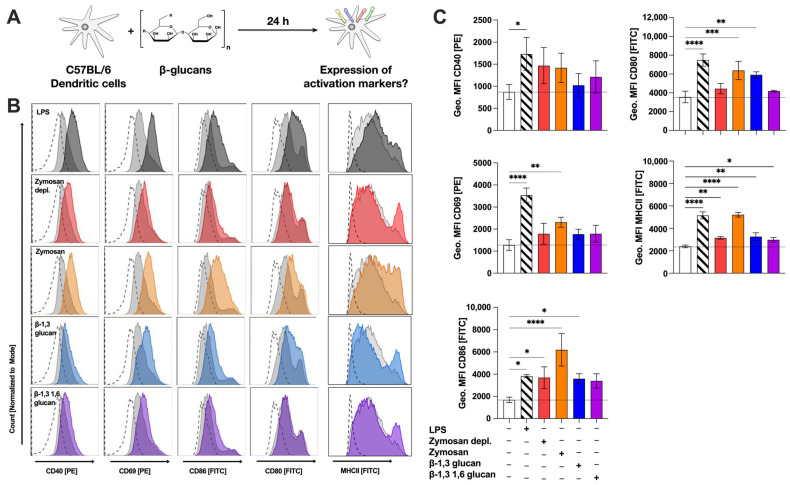
β-glucans upregulate the expression of MHCII; surface activation-, and co-stimulatory markers on mDCs. Bone marrow of C57BL/6 mice was isolated and differentiated for 8 days into mDCs that were subsequently stimulated with either 12 µg/mL of the indicated β-glucans or 10 µg/mL LPS as a positive control for 24 h (**A**). Cells were harvested, and surface expression of MHCII, the indicated activation markers, and co-stimulatory molecules were analyzed via flow cytometry. Stimulated samples (colored) were compared to either unstimulated controls (light grey filled) or fluorescence-minus-one (FMO)-stained cells (dashed lines). Data are representative results from one out of three independent experiments (**B**) or quantified as geometric mean fluorescence intensities (Geo. MFI) from three independent experiments (**C**). Dashed lines indicate the expression level of the unstimulated control. Statistical comparison was performed by 1-way ANOVA with correction for multiple comparisons according to Dunnett and indicated as follows: no indication = not significant and *p*-value > 0.05, * *p*-value < 0.05, ** *p*-value < 0.01, *** *p*-value < 0.001, or **** *p*-value < 0.0001. Abbreviations: MFI: mean fluorescence intensity.

To further characterize the mDC-activating properties of the different β-glucans, we next investigated the expression levels of MHCII, activation markers, and co-stimulatory molecules on β-glucan-stimulated CD11c^+^CD11b^+^B220^−^ mDCs by flow cytometry (Figure 4A).

All tested β-glucans slightly increased expression of the co-stimulatory molecule CD40 (Figure 4B), which, however, did not reach statistical significance (Figure 4C). While all tested β-glucans slightly increased expression levels of CD69, a significantly increased expression of CD69 was only observed for Zymosan-stimulated mDCs (MFI: 2311 compared to MFI 1277 for the unstimulated control, Figure 4B,C). CD86 expression was enhanced after stimulation with all tested β-glucans with Zymosan-stimulation inducing the highest geometric MFI levels (MFI: 6187), which were 1.6 times higher than those observed for the positive control LPS (MFI: 3820, Figure 4B,C). CD80 expression was significantly enhanced after stimulation with either Zymosan (MFI: 6374) or β-1,3 glucan (MFI: 5912) compared to the unstimulated control (MFI: 3560) (Figure 4B,C). Finally, MHCII expression was significantly increased by all tested β-glucans (Figure 4B,C). Here, Zymosan (MFI: 5215) induced the highest overall MHCII expression levels while Zymosan depl. (MFI: 3155), β-1,3 glucan (MFI: 3265), and β-1,3 1,6 glucan (MFI: 2979) increased MHCII expression only in a subset of mDCs, resulting in moderate overall increases compared to the unstimulated control (MFI: 2392) (Figure 4C).

### 2.6. β-Glucan-Induced Cytokine Secretion in Part Depends on Syk-Activation

**Figure 5:**
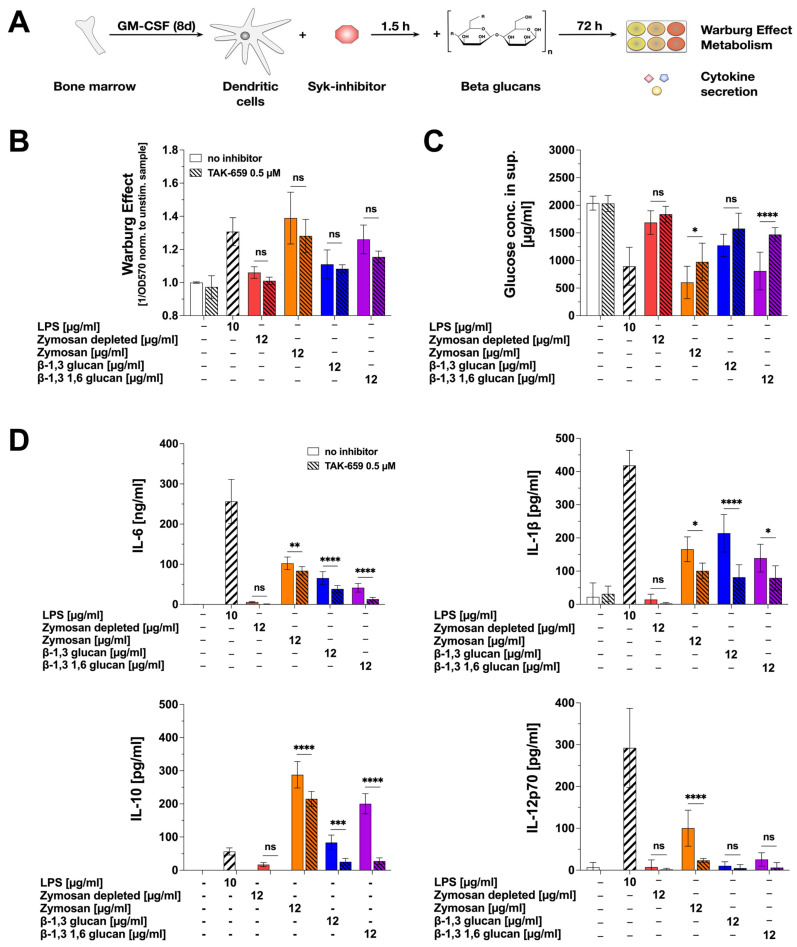
β-glucan-induced cytokine secretion in part depends on Syk-activation. Bone marrow of C57BL/6 mice was isolated, differentiated into mDCs for 8 days, pre-treated with 0.5 µM of the Syk-inhibitor TAK-659 for 90 min, and subsequently stimulated with either 12 µg/mL of the indicated β-glucans or 10 µg/mL of LPS as a positive control for additional 72 h (**A**). Bars with solid filling: stimulation without inhibitor pre-treatment, dashed bars: pre-treatment with 0.5 µM TAK-659 followed by the indicated stimulation. The Warburg Effect was measured at OD_570nm_, and the inverted values were normalized to the unstimulated controls (**B**). The glucose concentration in the cell culture supernatant was determined by using the Glucose (GO) assay kit and measuring the absorption at OD_540nm_ (**C**). The secretion of the cytokines IL-6, IL-1β, IL-10, and IL-12p70 was measured via sandwich ELISA at OD_450nm_ (**D**). Data are mean results of three independent experiments. Statistical comparison was performed by 2-way ANOVA with correction for multiple comparisons according to Dunnett and indicated as follows: ns = not significant and *p*-value > 0.05, * *p*-value < 0.05, ** *p*-value < 0.01, *** *p*-value < 0.001, or **** *p*-value < 0.0001.

In order to investigate the mechanisms contributing to the observed β-glucan-mediated mDC activation, we inhibited Dectin-1-mediated signaling using the Syk-inhibitor TAK-659 (Figure 5A).

The effects of the Syk-inhibitor on mDC metabolism were modest (Figure 5B,C): no significant effects were observed on the β-glucan-induced Warburg Effect, while Zymosan- and β-1,3 1,6 glucan-induced glucose consumption was slightly reduced compared to non-inhibited samples. When investigating β-glucan-induced cytokine secretion, TAK-659 slightly but significantly reduced β-glucan-induced IL-6 and IL-1β secretion (Figure 5D). Moreover, β-glucan-induced IL-10 secretion was suppressed by pre-treatment with TAK-659 (Zymosan 25.3%, Zymosan depl.: 100%, β-1,3 glucan: 69.66%, and β-1,3 1,6 glucan: 86.21% reduction compared to non-inhibited samples stimulated with the respective β-glucans). Zymosan-induced IL-12p70 production was strongly reduced by Syk inhibition (76.67% reduction compared to non-inhibited samples stimulated with Zymosan, Figure 5D).

In summary, our results suggest the β-glucan-induced activation of glucose metabolism to be largely Syk-independent, while cytokine secretion was in part dependent on Syk-activation.

### 2.7. β-Glucan-Stimulated mDCs Can Suppress IL-5 Production from Bet v 1-Specific CD4^+^ T Cells

**Figure 6:**
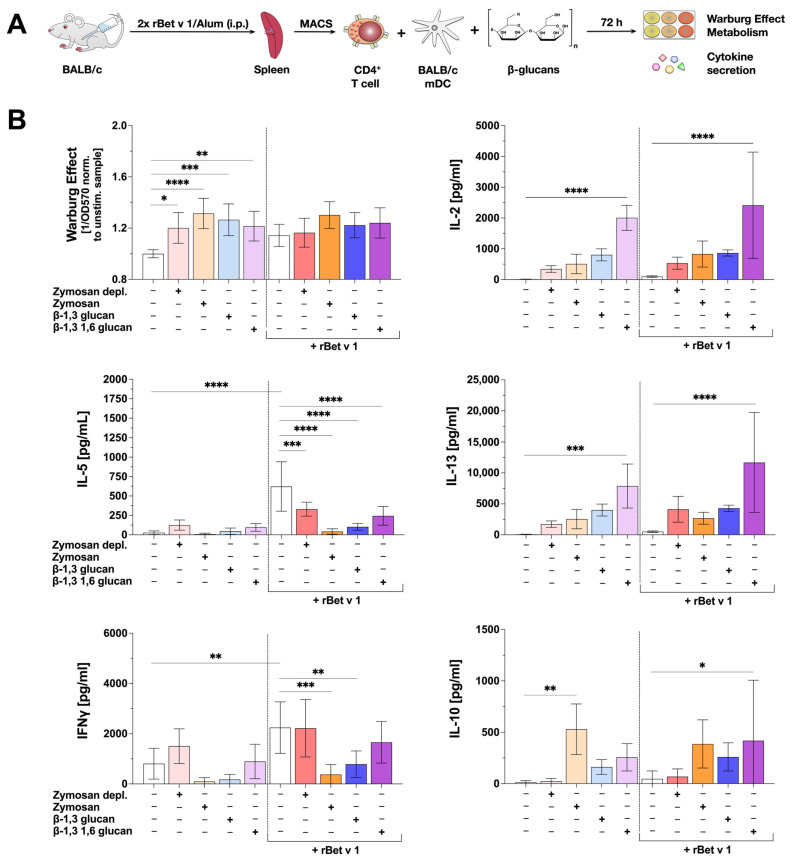
β-glucan-stimulated mDCs can suppress both IL-5 and IFNγ production from Th2-primed Bet v 1-specific CD4^+^ T cells. Bone marrow of BALB/c mice was isolated and differentiated into mDCs for 8 days. CD4^+^ T cells were isolated from BALB/c mice that were previously sensitized twice with 10 µg of the major birch pollen allergen Bet v 1 and 2 mg Alum i.p. The differentiated mDCs and the isolated T cells were co-cultured in 48-well plates and either stimulated with 8 µg of the respective β-glucan (lighter colors) or re-stimulated with 4.3 µg Bet v 1 in the presence of 8 µg of the respective β-glucan (darker colors) for additional 72 h (**A**). The Warburg Effect was measured at OD_570nm_ normalized to the unstimulated controls with or without Bet v 1, respectively (**B**). Secretion of IL-2, IL-5, IFNγ, IL-10, and IL-13 were determined via sandwich ELISA. Data are mean results ± SD of three independent experiments. Statistical comparison was performed by 1-way ANOVA with correction for multiple comparisons according to Dunnett and indicated as follows: no indication = not significant and *p*-value > 0.05, * *p*-value < 0.05, ** *p*-value < 0.01, *** *p*-value < 0.001, or **** *p*-value < 0.0001.

To examine the influence of β-glucan-mediated mDC activation on their T cell priming capacity, BALB/c mDCs were co-cultured with allergen-specific, Th2-biased CD4^+^ T cells isolated from spleens of BALB/c mice which were previously sensitized twice intraperitoneally (i.p.) with the major birch pollen allergen Bet v 1 using Alum as an adjuvant (37) (Figure 6A).

To study the effect of β-glucans on the allergen-induced secretion of Th1 and Th2 cytokines, the co-cultures were stimulated with the tested β-glucans with or without additional Bet v 1 re-stimulation for 72 h. The Warburg Effect and changes in allergen-induced cytokine secretion were measured (Figure 6A).

In co-cultures without Bet v 1 re-stimulation all tested β-glucans significantly increased the Warburg Effect. In contrast, in co-cultures with Bet v 1 re-stimulation, the Warburg Effect after stimulation with all tested β-glucans was not statistically different from co-cultures stimulated with Bet v 1 alone (Figure 6B). All tested β-glucans induced the secretion of the Th1 cytokine IL-2, compared to the unstimulated control (Figure 6B). However, this β-glucan-induced increase in IL-2 secretion was only statistically significant in co-cultures stimulated with β-1,3 1,6 glucan (either with or without Bet v 1 re-stimulation, Figure 6B).

Interestingly, the re-stimulation of the co-cultures with Bet v 1 resulted in a significant secretion of the Th2 cytokine IL-5 (Figure 6B). Additional stimulation with all tested β-glucans significantly suppressed the Bet v 1-induced IL-5 secretion by 46.8% (Zymosan depl.), 93.1% (Zymosan), 83.5% (β-1,3 glucan), or 60.7% (β-1,3 1,6 glucan), respectively (Figure 6B). Secretion of the Th2 cytokine IL-13 was induced by all tested β-glucans independent of the presence of Bet v 1 (as was the case for IL-2 only significantly for β-1,3 1,6 glucan, Figure 6B). Moreover, Zymosan and β-1,3 glucan (but not Zymosan depl. or β-1,3 1,6 glucan) significantly suppressed Bet v 1-induced IFNγ secretion by 75% and 42%, respectively (Figure 6B).

In co-cultures both with and without Bet v 1 re-stimulation, IL-10 production was induced by Zymosan, β-1,3 glucan, and β-1,3 1,6 glucan (Figure 6B). However, IL-10 levels were heterogenous, often not reaching statistical significance compared to unstimulated/Bet v 1-re-stimulated controls (Figure 6B).

Taken together, while all tested β-glucans suppressed Bet v 1-induced IL-5 production in co-cultures of mDCs with T cells, only Zymosan and β-1,3 glucan significantly suppressed Bet v 1-induced IFNγ secretion, suggesting the tested β-glucans to have distinct effects on the T cell priming capacity of mDCs.

## 3. Discussion

β-glucans are promising adjuvant candidates as they can promote anti-microbial activity, phagocytosis, and cytokine secretion in immune cells [8,9]. To better understand which β-glucans might be suitable as adjuvants, e.g., in allergen-specific immunotherapy, we directly compared six different β-glucans regarding their capacity to activate bone marrow-derived mouse mDCs and the effect of mDCs activated by the β-glucans Zymosan, Zymosan depl., β-1,3 glucan, or β-1,3 1,6 glucan on allergen-specific T cell responses.

### 3.1. β-Glucans Differ in Their Capacity to Trigger mDC-Derived Cytokine Secretion

The tested β-glucans triggered the production of both pro- and anti-inflammatory cytokines from mDCs with pronounced differences in β-glucan-mediated activation between the tested β-glucans: Zymosan induced the highest production of IL-6, IL-1β, IL-10, and IL-12p70 compared to the other tested β-glucans. The results are in accordance with reports that Zymosan induced the secretion of IL-10 in human and mouse DCs and TGF-β secretion in human and mouse macrophages [24], suggesting the induction of regulatory/tolerogenic APCs. Concerning Zymosan-induced IL-10 secretion, these results have been confirmed in the present study. Additionally, we demonstrated that Zymosan also induces the secretion of pro-inflammatory cytokines, such as IL-6, IL-1β, and IL-12p70. These results suggest that Zymosan co-induces both pro-inflammatory and regulatory immune responses. In direct comparison, mDC stimulation with Zymosan depl. resulted in strongly decreased cytokine secretion. Compared to Zymosan, β-1,3 glucan induced comparable IL-6, IL-1β, and IL-10 but lower IL-12p70 production, while for β-1,3 1,6 glucan, comparable production of IL-1β and IL-10 but only low levels of IL-6 and IL-12p70 were detected. Laminarin did not induce the secretion of any of the tested cytokines, whereas Curdlan-induced IL-1β-, and IL-10-production was lower compared to the other β-glucans. Depending on its physiochemical properties, purity, and structure, Laminarin was already described to show varying immune-modulatory properties, acting as Dectin-1 agonist or -antagonist [21,25,26]. Even while not inducing a pronounced cytokine secretion, the Curdlan preparation used in our experiments was characterized by a substantial endotoxin contamination (15,400 pg/µg β-glucan), which is likely caused by its extraction from the gram-negative bacterium *Alcaligenes faecalis* [27].

The β-glucans investigated in this study are natural products that may contain contaminating molecules. We observed detectable amounts of glucose in the used Zymosan, Zymosan depl., Laminarin, and β-1,3 1,6 glucan preparations. Furthermore, Zymosan, and to a lesser extent Zymosan depl. and Laminarin, contained proteins that could be detected by either SDS-PAGE or BCA/Bradford assays. While we do not expect the contained glucose to have immune-stimulating effects, a potential contribution of the protein fraction to the observed immune activation cannot be excluded. However, in our experimental system, Laminarin, which contained the highest glucose levels and detectable protein content, did not result in detectable mDC activation.

The comparison of endotoxin amounts contained within the 12 µg/mL of the β-glucans used for stimulation showed strongly different overall endotoxin levels (β-1,3 glucan: 27.36 pg/well, Zymosan depl.: 95.76 pg/well, Laminarin: 103.2 pg/well, Zymosan: 602.4 pg/well, β-1,3 1,6 glucan: 4164 pg/well, and Curdlan: 184,800 pg/well;). For the used mDC assay system, we established the threshold of LPS-induced mDC cytokine secretion to be approx. 1000 pg per well for IL-1β, approx. 100 pg for IL-6, and approx. 500 pg for IL-10 [28,29]. These results suggest that the endotoxin levels contained within the investigated β-1,3 glucan, Zymosan depl., and Laminarin preparations have minor contributions to the observed mDC activation, while for Zymosan, β-1,3 1,6 glucan, and Curdlan, immune–metabolic effects of the contained endotoxins cannot be excluded. However, when comparing Curdlan-induced mDC activation to the other tested β-glucans with either low (β-1,3 glucan, Laminarin, Zymosan depl.) or medium (Zymosan) amounts of residual endotoxin, there was no correlation between increasing endotoxin content and immune–metabolic mDC activation.

Although we cannot exclude synergistic effects of the contained endotoxins upon co-application with the tested β-glucans, these results suggest the immune-activating effects of the β-glucans to be complex and not dominated by the potential immune-activating capacity of contained endotoxins.

In contrast to our results, Elder and colleagues showed Curdlan to induce secretion of IL-6 and IL-1β in human mDCs [30]. However, the DCs were stimulated with a notably higher dose of Curdlan (50 µg/mL) than tested in this study (20 µg/mL) [30]. As the endotoxin content of Curdlan used by Elder et al. was not reported, it also cannot be excluded that DC activation observed by Elder et al. was at least in part caused by contaminating endotoxins.

In line with β-glucan-induced cytokine secretion, we also observed the upregulation of activation and co-stimulatory molecules. Again, Zymosan induced the strongest upregulation of CD40, CD86, CD80, and MHCII. That β-glucans may induce expression of co-stimulatory molecules was described by Kim and colleagues for *Sparassis crispa*-derived β-1,3 glucan [31].

### 3.2. Zymosan Depl., Zymosan, β-1,3 Glucan, and β-1,3 1,6 Glucan Strongly Activate mDC Metabolism

In this study, all tested β-glucans except for Laminarin induced a pronounced Warburg Effect paralleled by increased glucose consumption, suggesting the stimulation of mDCs with the tested β-glucans to increase glycolysis. Metabolic flux assays using Agilent Seahorse technology confirmed Zymosan depl., Zymosan, β-1,3 glucan, and β-1,3 1,6 glucan to enhance glycolytic activity of mDCs, while Zymosan depl., Zymosan, and β-1,3 glucan also enhanced the OCR, suggesting increased mitochondrial respiration. Our results suggest that stimulation with Zymosan depl., Zymosan, or β-1,3 glucan increases the general metabolic activity of the investigated mDCs. The simultaneous increase in both ECAR and OCR induced by some of the tested β-glucans could also be explained by an oxidative burst in which reduction equivalents generated in the Krebs cycle are used to produce reactive oxygen species (ROS) via, e.g., the ETC [32]. In contrast to the other tested β-glucans, Laminarin and Curdlan did not increase ECAR or OCR in extracellular flux assays, indicating that both could not activate mDC metabolism.

Taken together, our data demonstrate a strong activation of mDCs by β-glucans, particularly by Zymosan. However, the observed differences in the activation of mDC metabolism and effector function between the tested β-glucans suggest differences in the mechanisms underlying mDC activation.

### 3.3. The Strong mDC Activation by Zymosan May Be the Result of Dual TLR2- and Dectin-1-Activation

Even though we cannot directly compare activation levels of TLR2 and Dectin1 (due to the application of different detection methods and unknown differences in receptor expression, ligand affinity, and efficacy of reporter gene expression), HEK293 cell-based reporter assays performed in this study revealed that the strongest activation of human Dectin-1a/b was observed for Zymosan and Zymosan depl., followed by β-1,3 glucan, and β-1,3 1,6 glucan. Laminarin and Curdlan induced the lowest levels of hDectin-1a/b activation. Pronounced TLR2-activation was observed for Zymosan while being low for the other tested β-glucans. One drawback of our study is the use of human Dectin 1a/b reporter cells as we did not have access to reporter cells expressing mouse Dectin-1. However, our data obtained with the human Dectin-1 receptor variants may already be representative of the mouse situation, as Dectin-1 is highly conserved between both species [10]. Moreover, previous studies have shown β-glucans to activate Dectin-1 similarly in human and mouse cells [33,34].

The observed differences in receptor activation may explain why Zymosan generally elicits stronger immune responses than the other tested β-glucans. TLR2-activation results in MyD88-dependent activation of NFκB- and MAPK-signaling [35,36]. The Zymosan-induced TLR2-signaling seems to complement the Dectin-1-mediated cytokine production and surface expression of co-stimulatory molecules. This effect was particularly evident when directly comparing Zymosan to its variant Zymosan depl., which lacks TLR2-activating capacity [37,38], as stimulation with Zymosan resulted in notably higher production of pro- and anti-inflammatory cytokines and expression of co-stimulatory molecules.

Inhibition of the intracellular Dectin-1-associated adapter molecule Syk with TAK-659 demonstrated the increase in Warburg metabolism to be largely Syk-independent, while β-glucan-induced cytokine secretion was significantly but not completely reduced. Interestingly, IL-10 secretion was more strongly reduced in β-1,3 glucan- and β-1,3 1,6 glucan-stimulated cells than in Zymosan-stimulated cells, suggesting Zymosan-mediated IL-10 secretion be in part mediated by TLR2-signaling. Overall, our results suggest additional signaling pathways to contribute to the overall immune repones induced by the tested β-glucans.

Interestingly, stimulation with Zymosan, Zymosan depl., β-1,3 glucan, and β-1,3 1,6 glucan increased the surface expression levels of Dectin-1, TLR2, and the CR3 subunit CD18, even when not activating TLR2 themselves, indicating that Dectin-1-signaling might influence TLR2-signaling (in theory, e.g., by increasing TLR2 expression and thereby the sensitivity of the cells toward potential TLR2-ligands). Accordingly, the following was demonstrated: (I) Dectin-1 and TLR2 interact with each other, reinforcing their signaling [39,40]; (II) Dectin-1-signaling alone is not sufficient for β-glucan-induced cytokine production from mouse macrophages, further supporting that Dectin-1 interacts with other signaling pathways [41].

Of note, Zymosan depl. induced a stronger activation of mDC metabolism than Zymosan, reflected by higher increases in both ECAR and OCR. Here, the loss of Zymosan-mediated TLR2-signaling in stimulations with Zymosan depl. may result in lower production of certain cytokines that limit overshooting mDC responses (e.g., IL-10, TGF-β, IL-35 [42,43,44]). Therefore, mDC metabolism would be more strongly activated by Zymosan depl., although it lacks the TLR2-activating potential of its parent molecule. This speculation is supported by a markedly decreased IL-10 secretion in mDCs stimulated with Zymosan depl. when compared to corresponding stimulations with Zymosan. Another reason for the different mDC-activating effects of the tested β-glucans could be differences in composition, contaminating immune-stimulating molecules, or structural differences concerning chain length, type, and degree of branching. The immunogenicity of β-glucans was recently demonstrated to depend on their size, as smaller β-glucans induced weaker cytokine production from human DCs than β-glucans with a higher molecular weight [30]. Moreover, β-glucans with a higher degree of branching were shown to elicit stronger immune responses than those with a lower degree of branching [3,45].

### 3.4. β-Glucans Suppress IL-5 Secretion from Bet v 1-Specific Th2-Biased CD4^+^ T Cells but Have Different Effects on Bet v 1-Induced IFNγ Secretion

As mDCs, among other antigen-presenting cells, control the induction and modulation of adaptive immune responses via the activation of T cells, the influence of β-glucan-stimulated mDCs on allergen-specific, Th2-biased CD4^+^ T cells was studied in mDC:T cell co-culture experiments. In allergic patients, a switch from allergy-promoting Th2-responses toward either regulatory responses or Th1-responses may re-establish tolerance toward the respective allergens [46].

In contrast to the above-described experiments using mDCs derived from C57BL6/N mice, co-cultures were performed using mDCs and T cells derived from BALB/c mice. Due to their genetic background, C57BL/6 mice are more prone to establishing Th1-responses and mount only weak IgE-responses [47]. Therefore, in order to study the effects of the investigated β-glucans on Th2 responses, we used cells from the Th2-prone BALB/c background for the performed co-cultures. DCs in BALB/c and C57BL/6 mice were reported to show differences in TLR expression and reactivity [48]. However, with the exception of a weaker IL-1β secretion induced by Zymosan and Zymosan depl. in BALB/c mDCs and a lower IL-6 secretion induced by β-1,3 1,6 glucan in C57BL/6N mDCs, we did not observe relevant differences in the β-glucan-induced cytokine profile between mDCs differentiated from both mouse strains, suggesting the β-glucan-induced activation of mDCs from both strains to be comparable.

Zymosan, Zymosan depl., β-1,3 glucan, and β-1,3 1,6 glucan all significantly suppressed Bet v 1-induced IL-5 production, while IFNγ was only significantly suppressed by Zymosan and β-1,3 glucan. Interestingly, suppression of Bet v 1-induced IFNγ production correlated with increased levels of IL-10 in co-cultures stimulated with Zymosan and β-1,3 glucan.

While Zymosan-stimulated mDCs could suppress Bet v 1-induced IFNγ secretion in mDC:T cell co-cultures, mDCs stimulated with Zymosan depl. had no effect on Bet v 1-induced IFNγ secretion. Therefore, our results suggest the suppression of the Bet v 1-induced IFNγ production to be mediated via TLR2. In line with this, we observed differences in mDC-derived cytokine production induced by Zymosan and Zymosan depl., e.g., the lower IL-10 production induced by the Zymosan depl. discussed above.

The potential of β-glucans to modulate T cell responses has already been demonstrated: C57BL/6 mouse mDCs stimulated with yeast-derived β-glucan promoted both Th1 differentiation and cytotoxic T cell responses [49]. Moreover, in an in vivo mouse tumor model, β-glucan treatment enhanced the secretion of IFNγ [50].

Furthermore, co-culture experiments showed Zymosan, Zymosan depl., β-1,3 glucan, and β-1,3 1,6 glucan to induce both IL-2 and IL-13 production independent of the presence of the allergen. Additionally, β-glucan-induced IL-2 and IL-13 levels were similar for cultures of mDCs and mDC:T cell co-cultures, suggesting the β-glucan-induced production of IL-2 and IL-13 to be derived from the mDCs in the co-culture. Reports of DCs producing IL-13 are rare, but Bellinghausen and colleagues in 2003 described human DCs stimulated with protein allergens to promote Th2 responses via the secretion of IL-13 [51].

While several publications have shown β-glucans to suppress Th2 inflammation, there are also some studies demonstrating the aggravation of Th2 responses by the application of β-glucans: Higher serum levels of IL-4 and IL-13 were reported in rabbits receiving a diet supplemented with β-glucans isolated from shiitake mushrooms [52]. Moreover, in addition to LPS, β-glucans contained in fine matter particles were suggested to exacerbate murine lung eosinophilia paralleled by increased IL-4 and IL-13 production in bronchoalveolar lavage fluid [53]. However, the cellular source of the IL-13 remained elusive in these studies. Finally, *Aspergillus fumigatus*-induced secretion of IL-13 from mouse-purified lung CD4^+^ T cells was shown to be in part dependent on Dectin-1 [54].

Our results showed β-glucan-stimulated mDCs to distinctly modulate allergen-specific T cell responses (e.g., suppression of Bet v 1-induced IL-5 secretion by the tested β-glucans or the suppression of Bet v 1-induced IFNγ production by Zymosan-stimulated mDCs). Further experiments are needed to understand, e.g., the contribution of the β-glucan-induced IL-13 production to the overall immune responses.

Among the tested β-glucans, Zymosan (strongly suppressing IL-5 and IFNγ production) and Zymosan depl. (suppression of IL-5 secretion with retained IFNγ production) appear to be the most promising adjuvant candidates for an application in allergen-specific immunotherapy. This is supported by the findings of Lee and colleagues, showing that Zymosan enhances Th1-responses by inducing IFNγ secretion in mouse DCs [4]. So far, no clinical trial reports the application of these β-1,3 glucans in allergen immunotherapy (AIT).

Also, β-1,3 glucan and β-1,3 1,6 glucan show the potential to partially suppress Bet v 1-induced IL-5 cytokine secretion while simultaneously promoting (IL-2) or maintaining (IFNγ) the secretion of Th1 cytokines. In line with these results, regular topical application of a 0.25% β-glucan-based cream (Imunoglukan P4H^®^ cream) in a non-industry-sponsored, multicenter open split-body study in 105 patients with atopic dermatitis reduced pruritus and severity of atopic dermatitis with only mild local side effects [55]. Furthermore, oral-administration of superfine dispersed β-(1,3)-glucan reduced pollen-specific IgE antibody levels in a phase IV clinical study (sponsored by the Meiji University of Oriental Medicine, Kyoto, Japan) [56]. The superfine dispersed β-(1,3)-glucan also alleviated rhinorrhea, sneezing, nasal congestion, and itchy, watery eyes otherwise induced by Japanese cedar pollen in allergic patients [56].

However, oral treatment with the same β-1,3 glucan (but not β-1,3 1,6 glucan) used in our study was recently shown to promote allergic responses in an OVA-based mouse intestinal allergy model [16], suggesting that this β-1,3 glucan might not be suitable for immunotherapy purposes.

Finally, in a non-industry-sponsored, randomized, doubled-blind, parallel group, and placebo-controlled study, an oral treatment with 10 mg β-(1,3-1,6)-glucan twice a day for 12 weeks significantly reduced levels of Th2 cytokines and eosinophils in nasal lavage fluid while increasing levels of IL-12 in 26 patients with seasonal allergic rhinitis (AR) sensitized to olive pollen [57].

Recently, 5-mer β-1,3 oligomers were shown to trigger IL-2, TNFα, and IFNγ mRNA expression in Jurkat T cells [58]. Atomistic molecular dynamics simulations suggested that the β-1,3 oligomers induce T cell activation via binding to CD28. The theoretical K_D_ for a 8-mer β-1,3 oligomer binding to CD28 was determined to be 1.1 mM [58]. In contrast to these results, we were unable to observe direct activation of CD4^+^ T cells with the tested β-glucans. This discrepancy may be caused by different factors: the longer chains of the β-glucans tested by us compared to the 5- to 8-mers in Comer et al. might be unable to bind to CD28, the usage of mouse vs. human cells, or the lack of anti-CD3 antibodies in our culture system.

### 3.5. Some β-Glucans Are Interesting Adjuvant Candidates for AIT

Taken together, by directly and comprehensively comparing the mDC-activating potential of six different β-glucans, we showed β-glucans to induce distinct immune responses in mouse myeloid mDCs. These differences in mDC activation may be caused by (I) differences in the abilities of the tested β-glucans to activate Dectin-1-, TLR2-, and CR3-dependent intracellular signaling cascades and (II) differences with regard to composition, contaminating immune-activating molecules, chain length, type, and degree of branching. Among the tested β-glucans, we identified Zymosan and Zymosan depl. to be promising adjuvant candidates for usage in AIT as they were able to strongly suppress allergen-induced IL-5 secretion while either suppressing (Zymosan) or retaining (Zymosan depl.) IFNγ production. Here, further studies are needed to gain a more complete picture of the immune-modulating properties of β-glucans.

## 4. Material and Methods

### 4.1. β-Glucans

Tested β-glucans Zymosan (tlr-zyn, batch no. 5933-43-01), Zymosan depl. (tlrl-zyd, batch no. 6294-44-01), Laminarin (tlr-lam, batch no. LMn-41-01), and Curdlan (tlrl-curd, batch no. 6291-43-01) were obtained from InvivoGen (Toulouse, France), β-1,3 glucan from Biosynth Carbosynth^®^ (Compton, UK, YG30829, batch no. 0000034085), and β-1,3 1,6-glucan from Oriental Yeast. Co., Ltd. (Tokyo, Japan, 82384-00, no batch no. available). β-glucans were solved in either PBS or PBS containing 0.1 M NaOH to a final concentration of 10 mg/mL. Stimulations of cells were performed with β-glucans solved in PBS only.

### 4.2. Endotoxin Determination

Endotoxin concentrations in the used β-glucan preparations were determined via chromogenic Limulus amebocyte lysate (LAL) test according to the manufacturer’s instructions (Charles River PTS LAL, Sulzfeld, Germany).

### 4.3. SDS-PAGE and Protein Determination

An amount of 50 µg of Zymosan, Zymosan depl., Laminarin, Curdlan, β-1,3 glucan, or β-1,3 1,6 glucan was diluted with 4X Laemmli sample buffer (Bio-Rad Laboratories, Hercules, CA, USA), followed by heat-inactivation at 95 °C for 5 min. Moreover, 15 µL (containing 112.5 µg of β-glucan) of each sample, 2.5 µL PageRuler™ plus Prestained Protein Ladder (Thermo Scientific, Rockford, IL, USA), or 5 µL Precision Plus Protein™ Dual Xtra Standards (Bio-Rad Laboratories) were loaded onto a Mini-PROTEAN^®^TGX™ Precast 4–20% gel (Bio-Rad Laboratories). The gel was stained using Coomassie^®^ brilliant blue G250 (Bio-Rad Laboratories).

Total protein concentration in β-glucan preparations was determined by both bicinchoninic acid (BCA, Pierce™ BCA Protein Assay Kits, Thermo Fisher Scientific, Sindelfingen, Germany) and Bradford assay (Pierce™ Bradford Protein Assay Kit, Thermo Fisher Scientific).

### 4.4. DC Preparation and Stimulation

C57BL/6N (Jackson Laboratories, Bar Harbor, ME, USA) mouse bone marrow-derived myeloid dendritic cells (mDCs) were differentiated using recombinant mouse granulocyte-macrophage-colony stimulating factor (GM-CSF) [28]. On day 8, loosely adherent cells were harvested, and either 8 × 10^4^ cells were seeded in a total volume of 200 µL in 96 well plates or 0.5 × 10^6^ cells were seeded in a total volume of 1 mL in 24 well plates. Cells were stimulated with either the indicated amounts of the β-glucans or lipopolysaccharide (LPS; L5886, Sigma Aldrich, Taufkirchen, Germany) as a positive control for the indicated durations.

### 4.5. Enzyme-Linked Immunosorbent Assay (ELISA)

Supernatants were analyzed for cytokine secretion by ELISA 72 h after stimulation using the following antibody combinations: IL-1β (either using Mouse IL-1β/IL-1F2 DuoSet ELISA (R&D Systems, Inc., McKinley Place, MN, USA, Figure 5) or capture antibody: IL-1β monoclonal mouse antibody 1:500 (#14-7012-85, eBioscience, Frankfurt, Germany) and detection antibody: IL-1β polyclonal mouse biotin-conjugated antibody 1:500 (#13-7112-81, eBioscience, all other figures)), IL-6 (capture antibody: IL-6 monoclonal mouse antibody 1:1000 (#14-7061-85, eBioscience) and detection antibody: IL-6 monoclonal mouse biotin-conjugated antibody 1:1000 (#13-7062-85, eBioscience)), IL-12 (capture antibody: purified anti-mouse IL-12p70 (clone: C18.2, 1:500) and detection antibody biotin anti-mouse IL-12p70 (clone: C17.8, 1:500)). Levels of IL-10 were measured using the mouse ELISA Development Kit from PeproTech (#900-T53, Pepro-Tech, Thermo Fisher Scientific, Hamburg, Germany) according to the manufacturer’s recommendations.

### 4.6. Detection of the Warburg Effect and Glucose Consumption in Cell Culture Supernatants

Supernatants of the experiments were harvested 72 h after stimulation and measured with a SpectraMaxPlus384 microplate reader (Molecular Devices, San José, CA, USA). As there is so far no systematic approach to define, quantify, or analyze the Warburg Effect [59], we used a self-developed method to quantify the Warburg Effect by measuring the OD_570nm_ values, inverting them, and normalizing them to the respective unstimulated control samples. Glucose concentrations in the harvested cell culture supernatants 72 h post-stimulation were determined using the Glucose (GO) assay kit (Sigma-Aldrich) [60].

### 4.7. Extracellular Flux Assays

For metabolic flux analysis, 1 × 10^5^ mDCs per well were seeded in Seahorse XF96 cell culture microplates (V3-PS, tissue culture-treated, Agilent, Santa Clara, CA, USA). The next day, the medium was exchanged, and cells were stimulated as indicated with the different β-glucans. Seahorse XF Real-Time ATP rate assays were performed according to the manufacturer’s recommendations (Agilent). Cycle numbers were as follows: four cycles of baseline measurement, 14 cycles of stimulation with the different β-glucans, and 8 cycles with oligomycin, rotenone/antimycin A (Rot/AA), or 2-deoxy-glucose (2-DG), respectively (1 cycle = 3 min mixing plus 3 min measuring). Post measurement, samples were normalized to total protein content via bicinchoninic acid (BCA) assay (Thermo Fisher Scientific) and analyzed using Wave Desktop Software version 2.6.3.8 (Agilent) and GraphPad Prism version 9.2.0. for MacOS and Windows (GraphPad Software Inc., San Diego, CA, USA).

### 4.8. Flow Cytometry

For flow cytometric analyses, mDCs were seeded at 5 × 10^5^ cells/mL in 24-well plates (Thermo Fisher Scientific) and stimulated with the indicated β-glucan concentrations for 24 h. Moreover, 10 µg/mL LPS (L5886, Sigma Aldrich, Taufkirchen, Germany) served as a positive control. The phenotype of CD11b^+^CD11c^+^B220^−^ mDCs was assessed using the following anti-mouse antibodies: either (I) Pacific Blue-conjugated CD11b (clone: M1/70.15, dilution: 1:50; Invitrogen, Thermo Fisher Scientific, Schwerte, Germany), phycoerythrin cyanine 5.5 (PE-Cy5.5)-conjugated CD11c (clone: N418, dilution: 1:100; Invitrogen, Thermo Fisher Scientific), and PE-Cy5-conjugated B220 (clone: RA3-6B2, dilution: 1:500; BD Biosciences, Heidelberg, Germany) or (II) PE-Cy7-conjugated CD11b (clone: M1/70, dilution: 1:50; Invitrogen, Thermo Fisher Scientific), eFlour^TM^450-conjugated CD11c (clone: N418, dilution: 1:100; Invitrogen, Thermo Fisher Scientific), and PE-Cy5-conjugated B220 (clone: RA3-6B2, dilution: 1:100; Invitrogen, Thermo Fisher Scientific). β-glucan-induced expression of pattern recognition receptors was analyzed using anti-mouse antibodies: PE-conjugated mDectin-1 (clone: 2A11, dilution: 1:10; Invitrogen, Thermo Fisher Scientific), fluorescein isothiocyanate (FITC)-conjugated mTLR2 (clone: 6C2, dilution: 1:50; Invitrogen, Thermo Fisher Scientific), or PE-conjugated mCD18 (clone: M18/2, dilution: 1:25, Thermo Fisher Scientific). Activation of mDCs was analyzed using anti-mouse antibodies: PE-conjugated CD40 (clone: 1C10, dilution: 1:10; Invitrogen, Thermo Fisher Scientific), PE-conjugated CD69 (clone: H1.2F3, dilution: 1:150; Invitrogen, Thermo Fisher Scientific), FITC-conjugated CD86 (clone: B7-02, dilution: 1:50; Invitrogen, Thermo Fisher Scientific), FITC-conjugated CD80 (clone: 16-10A1, dilution: 1:50; Invitrogen, Thermo Fisher Scientific), or FITC-conjugated MHCII (clone: M5, dilution: 1:100, eBioscience). Intensities of PE or FITC of CD11b^+^CD11c^+^B220^−^ cells (mDCs) were measured using a BD Symphony flow cytometer (BD Biosciences). Geometrical mean fluorescence intensities were calculated and normalized to unstimulated cells. Data were analyzed using FlowJo V.7 (Treestar Inc., Ashland, OR, USA).

### 4.9. Reporter Assays to Determine Receptor Activation

HEK-Blue^™^ reporter cell lines stably transfected with either human Dectin-1a/b (InvivoGen, Toulouse, France) or HEK-293 reporter cells stably transfected with mouse TLR2 (all commercially available at InvivoGen) were cultured in Dulbeccos Modified Eagle Medium (DMEM) (Gibco, Paisley, UK) containing 10% fetal calf serum (FCS), 1% streptomycin, penicillin, L-glutamine (SPL), and the respective selection antibiotics according to the manufacturer’s recommendations. For Dectin-1 activation assays, 5 × 10^4^ cells/well were seeded in 96-well plates and stimulated with increasing doses of the β-glucans for 16 h. Subsequently, the OD_635nm_ value was measured, and the amount of SEAP was determined and normalized to the unstimulated controls. For TLR2 activation assays, 5 × 10^4^ cells/well were seeded in 96-well plates in DMEM containing 2% FCS and stimulated with increasing doses of the β-glucans for 24 h. Afterward, IL-8 secreted in the supernatant was quantified using the human OptEIA^TM^ IL-8 ELISA Set (BD Biosciences, Heidelberg, Germany).

### 4.10. Inhibition Experiments

In 24 well plates, 5 × 10^5^ C57BL/6N mDCs were seeded in a total volume of 0.5 mL. Cells were pre-treated with 0.5 µM of the Syk inhibitor TAK-659 (Selleckchem, Thermo Fisher Scientific, Schwerte, Germany). 90 min later, cells were stimulated with the indicated amounts of the β-glucans, wells were filled to a total volume of 1 mL, and incubated for additional 72 h at 37 °C and 5% CO_2_. Supernatants were analyzed for the induction of the Warburg Effect and cytokine secretion. Toxicity of TAK-659 on mDCs was determined using the fixable viability dye eFlour™780 (ThermoFisher Scientific). Inhibitor concentrations showing toxic effects were excluded from the analyses.

### 4.11. mDC: T Cell Co-Cultures

BALB/c (Jackson Laboratories, Bar Harbor, ME, USA) mDCs (5 × 10^5^ cells/mL) were cultured in 48-well plates with splenic CD4^+^ T cells (6.3 × 10^5^ cells/mL) isolated from BALB/c mice sensitized with Bet v 1 plus aluminum hydroxide (Alum) (2 times 10 µg Bet v 1 plus 0.5 mg Alum i.p., 2 weeks apart, approval number: F107/1049, Regierungspräsidium Darmstadt) using the CD4^+^ T Cell Isolation Kit (Miltenyi Biotech, Bergisch Gladbach, Germany). Cultures were stimulated with either Bet v 1 or Bet v 1 plus the indicated β-glucans for 72 h. Subsequently, cytokine secretion into culture supernatants was determined using either BD OptEIA™ ELISA Sets (IFNγ, IL-5, BD Bioscience), Ready-Set-Go-ELISA kits (IL-13, eBioscience) or the following antibody combination for IL-2: capture antibody: IL-2 monoclonal anti-mouse antibody 1:500 (#503702, BioLegend, Koblenz, Germany) plus detection antibody: IL-2 monoclonal anti-mouse biotin-conjugated antibody 1:500 (#503804, BioLegend).

### 4.12. Statistical Analyses

Statistical analyses were performed using GraphPad Prism version 9.2.0 for MacOS and Windows. Data were tested using either 1-way or 2-way Analysis of variance (ANOVA) tests, which were adjusted for multiple comparisons according to either Dunnett or Bonferroni. For statistically significant results, the following convention was used: * *p*-value < 0.05, ** *p*-value < 0.01, *** *p*-value < 0.001, or **** *p*-value < 0.0001.

## Data Availability

The data presented in this study are available on request from the corresponding author.

## Supplementary Materials

The following supporting information can be downloaded at: https://www.mdpi.com/article/10.3390/ijms25189914/s1.

## Abbreviations

2-DG	2-deoxyglucose
ATP	adenosine triphosphate
Alum	aluminum hydroxide
BCA	bicinchoninic acid
CD[X]	cluster of differentiation [X]
CLR	C-type lectin
ECAR	extracellular acidification rate
ELISA	enzyme-linked immunosorbent assay
ETC	electron transport chain
FMO	fluorescence-minus-one
GM-CSF	granulocyte-macrophage-colony stimulating factor
HEK	human embryonic kidney
IL-[X]	interleukin-[X]
IFNγ	interferon gamma
i.p.	intraperitoneal
LAL	limulus amebocyte lysate
LPS	lipopolysaccharide
mDC	myeloid dendritic cell
Geo. MFI	geometric mean fluorescence intensity
OCR	oxygen consumption rate
OD	optical density
PBS	phosphate-buffered saline
PRR	pattern recognition receptor
ROS	reactive oxygen species
Rot/AA	rotenone/antimycin A
SEAP	secreted embryonic alkaline phosphatase
Th1/2	T helper cell type 1/2
TLR	“Toll”-like receptor
