# Supplementary material for: Characterization of the Immune-Modulating Properties of Different β-Glucans on Myeloid Dendritic Cells
Source: Int J Mol Sci. 2024 Sep 13;25(18):9914. doi: 10.3390/ijms25189914 (PMC11433108; doi:10.3390/ijms25189914)
Supplement: Supplementary file 1 [file ijms-25-09914-s001.zip › ijms-3195119-supplementary.pdf]

Supplemetary Materials

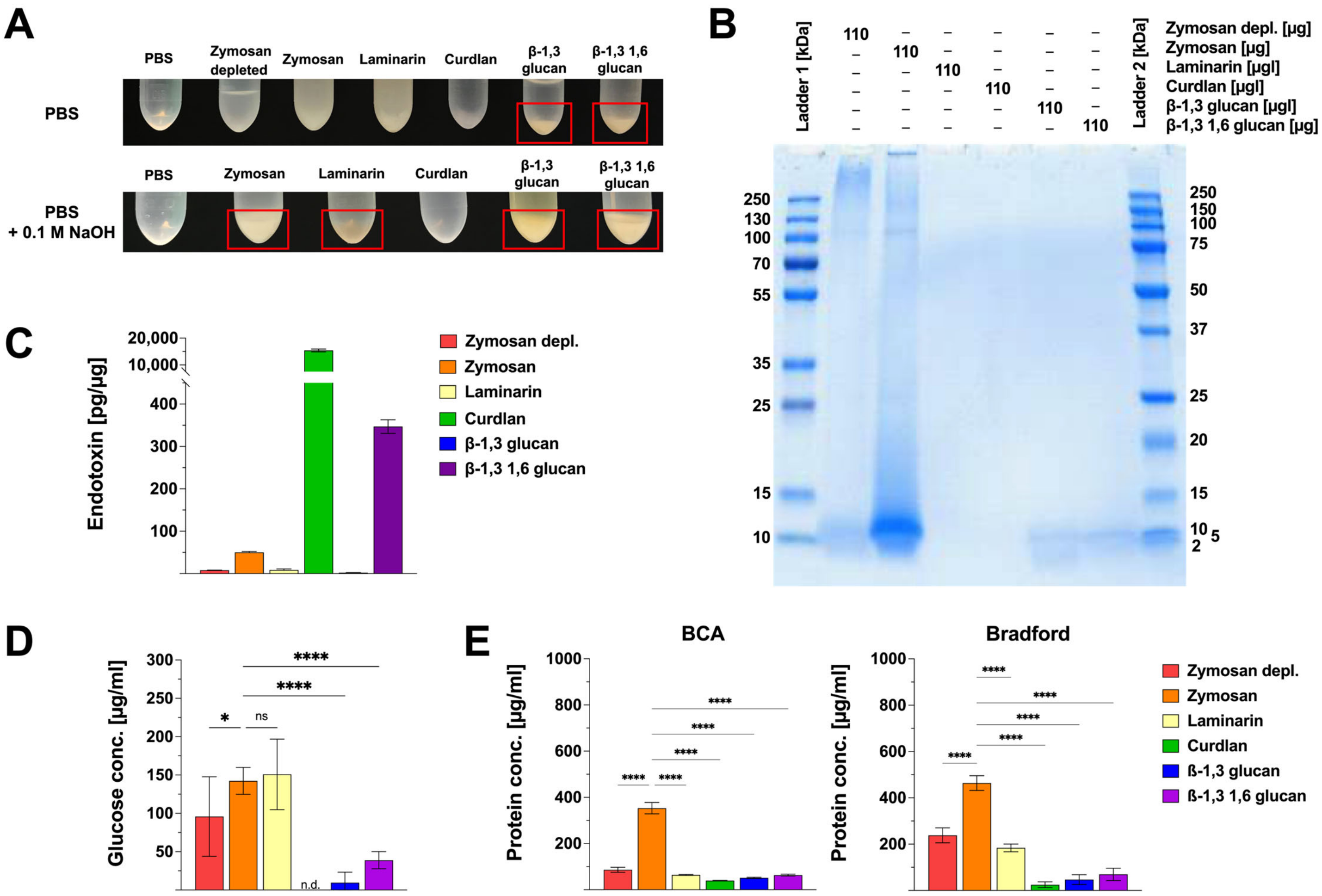

Figure S1. β-glucans show different solubilities, endotoxin levels, protein- and glucose content. The tested β-glucans were solubilized in PBS and their solubility was examined visually (**A**). Proteins contained within the β-glucan preparations were separated by SDS-PAGE. Ladder 1: 2.5 µl PageRuler™ Plus Prestained Protein Ladder,

ladder 2: 5 µl Precision Plus Protein™ Dual Xtra Standard (**B**). Endotoxin levels of the different β-glucans were determined using the Charles River Endosafe® LAL cartridge technology. Data are mean results ± CV [pg/µg] of four technical replicates (**C**). Glucose concentration (determined using the Glucose (GO) Assay kit and measured at OD_{540nm} (**D**), and total protein concentration, determined via either BCA or Bradford assay (**E**) in the β-glucans preparations was investigated. Data are mean results of three independent measurements (**D&E**). Statistical analysis was performed using 1-way ANOVA with corrections for multiple comparisons according to Dunnett and indicated as: ns = not significant and p-value > 0.05, * p-value < 0.05 or **** p-value < 0.0001. Abbreviations: n.d. = not detectable.

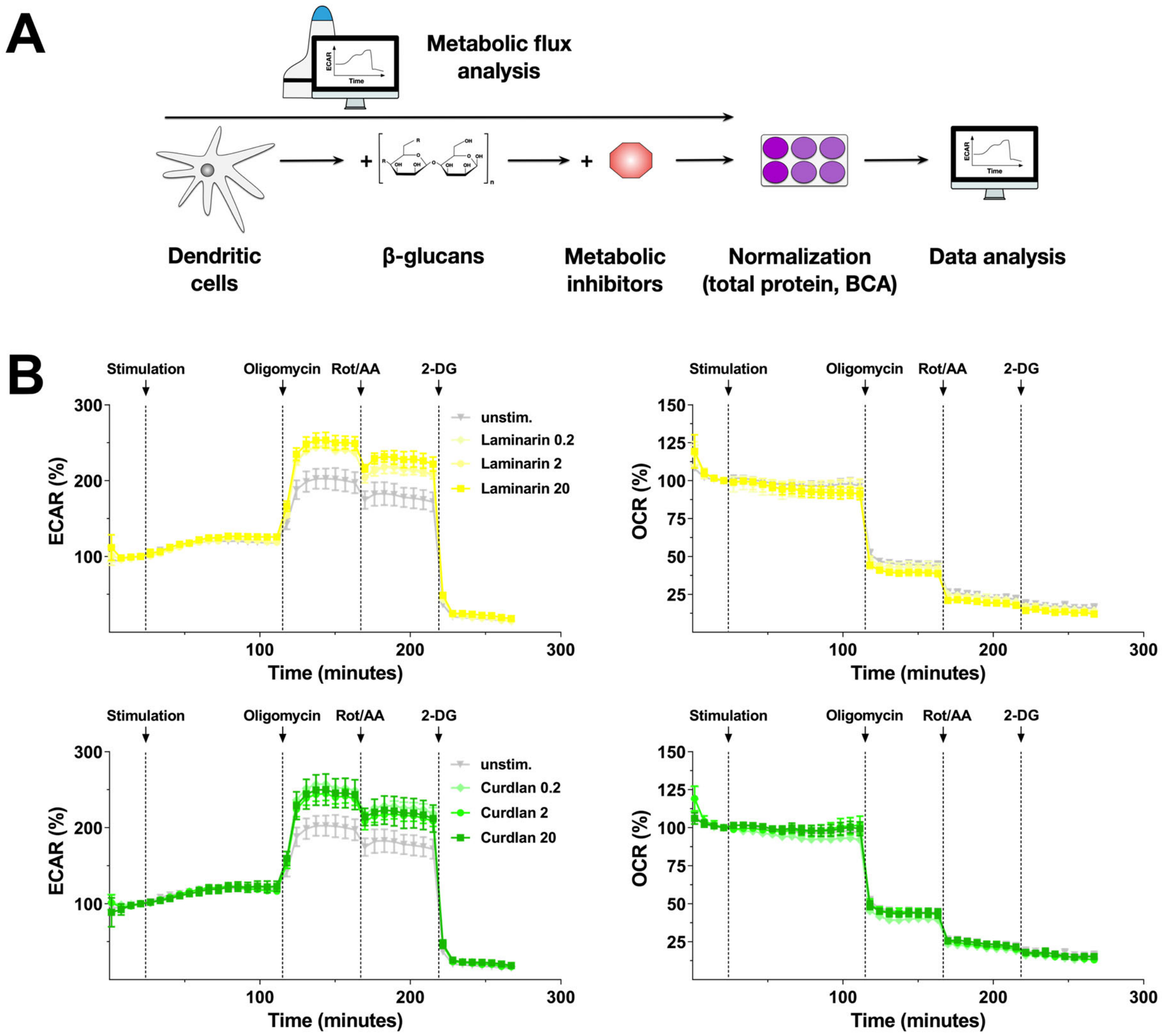

Figure S2. Laminarin and Curdlan do not activate mDC metabolism. Bone marrow of C57BL/6 mice was isolated, differentiated into mDCs for 8 days, and subsequently analyzed in extracellular flux assays using Agilent Seahorse Technology (**A**). mDCs were seeded overnight into Seahorse XF96 cell culture microplates,

stimulated with increasing doses of the indicated β-glucan for 14 cycles (84 min), and analyzed for ECAR and OCR. Afterwards, ATP synthase, electron transport chain, and glycolysis were inhibited by sequentially injecting oligomycin, Rotenone/antimycin A (Rot/AA), and 2-deoxyglucose (2-DG), respectively for 8 cycles (48 min) each. Data are representative of three independent experiments (**B**). Abbreviations: ECAR: extracellular acidification rate, OCR: oxygen consumption rate, Rot/AA: rotenone/antimycin A, 2-DG: 2-deoxyglucose.

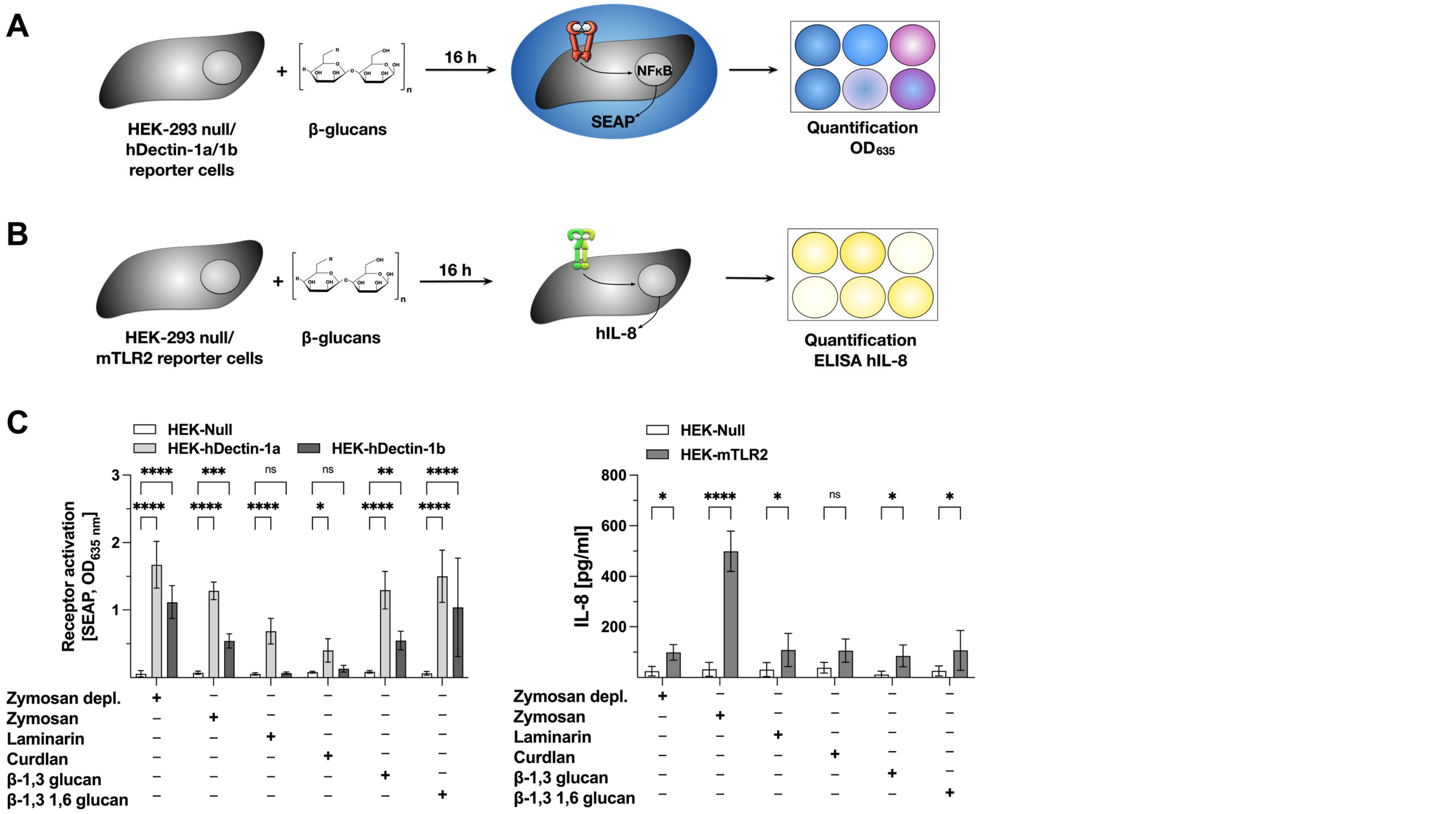

Figure S3. β-glucans differentially activate the PRRs TLR2 and Dectin-1. HEK-Blue™ reporter cells stably transfected with human (h)Dectin-1a or 1b (**A**,**C**) or HEK-293 reporter cells stably transfected with murine (m)TLR2 (**B**,**C**) were stimulated for 16 h with increasing doses of the indicated β-glucans ranging from 0.001 μg/ml to 500

µg/ml. Dectin-1a/b activation was quantified by detecting the secreted alkaline phosphatase (SEAP) at OD_{635nm} (**A,C**) and TLR2 activation by determining the production of human IL-8 by ELISA (**B,C**). Data are mean results ± SD from three independent experiments. For statistical comparison, the stimulation concentration of 100 µg/ml for all tested β-glucans was compared between the different cell lines (**C**). Statistical comparison was performed by 2-way ANOVA with correction for multiple comparisons according to Bonferroni and indicated as: ns = not significant and *p*-value > 0.05, * *p*-value < 0.05, ** *p*-value < 0.01, *** *p*-value < 0.001, or **** *p*-value < 0.0001.

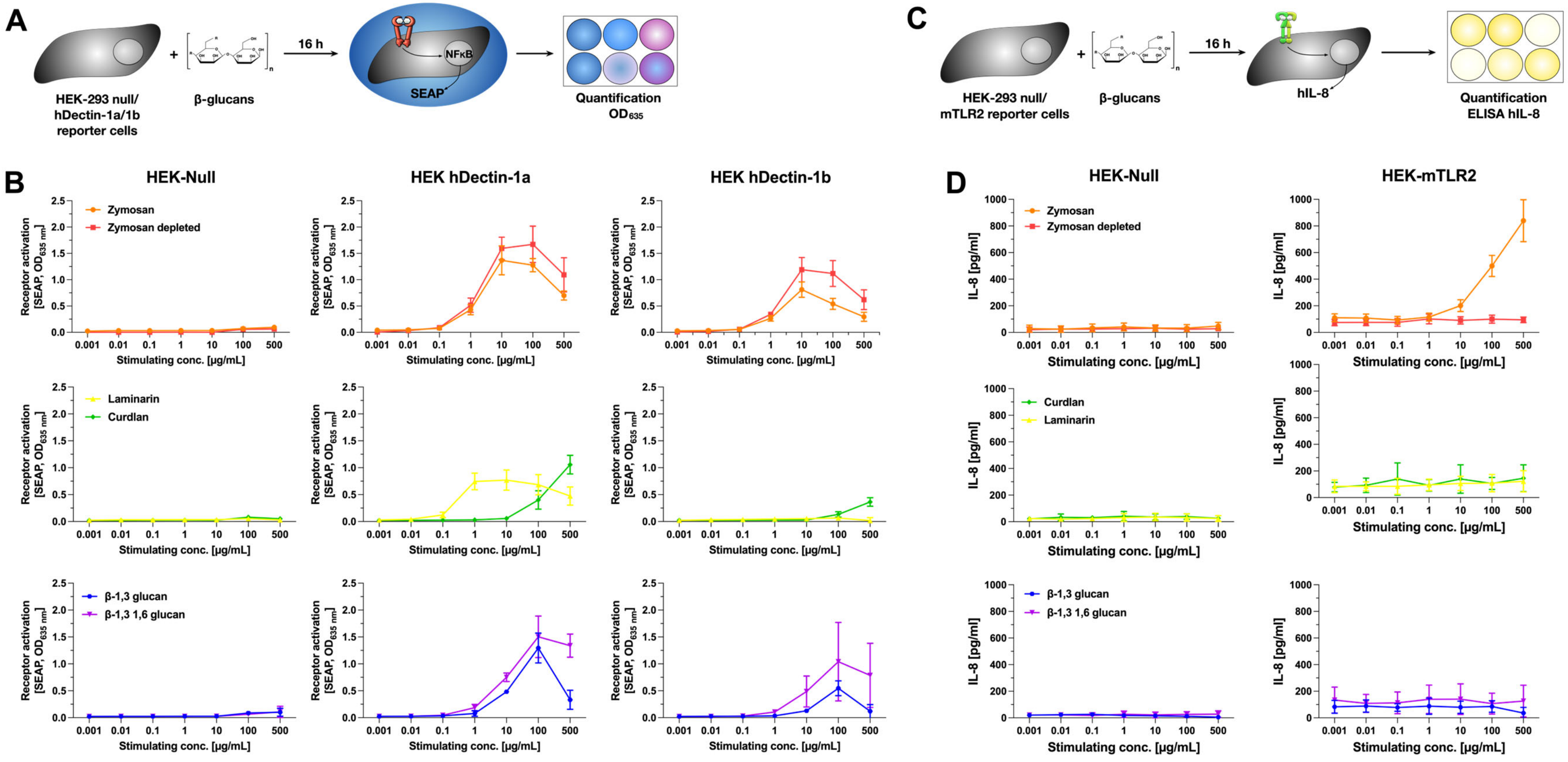

Figure S4: β-glucans differentially activate the PRRs TLR2 and Dectin-1. HEK-Blue™ reporter cells stably transfected with human (h)Dectin-1a or 1b (**A,B**) or HEK-293 reporter cells stably transfected with murine (m)TLR2 (**C,D**) were stimulated for 16 h with increasing doses of the indicated β-glucans ranging from 0.001 µg/ml

to 500 μg/ml. Dectin-1a/b activation was quantified by detecting the secreted alkaline phosphatase (SEAP) at OD_{635nm} (**A,B**) and TLR2 activation by determining the production of human IL-8 by ELISA (**C&D**). Data are mean results ± SD from three independent experiments.
